# Association of Human Leukocyte Antigen Class 1 genes with Stevens Johnson Syndrome with severe ocular complications in an Indian population

**DOI:** 10.1038/s41598-017-15965-7

**Published:** 2017-11-21

**Authors:** Chitra Kannabiran, Mayumi Ueta, Virender Sangwan, Varsha Rathi, Sayan Basu, Katsushi Tokunaga, Shigeru Kinoshita

**Affiliations:** 1 Kallam Anji Reddy Molecular Genetics Laboratory, Prof Brien Holden Eye Research Centre; Tej Kohli Cornea Institute, Hyderabad, India; 2 Department of Frontier Medical Science and Technology for Ophthalmology, Kyoto Prefectural University of Medicine, Kyoto, Japan; 3 L.V. Prasad Eye Institute, Kallam Anii Reddy Campus, Banjara Hills, Hyderabad, 500034 India; 4 Department of Human Genetics, Graduate School of Medicine, University of Tokyo, Tokyo, Japan

## Abstract

Stevens Johnson syndrome (SJS) is part of a spectrum of adverse drug reactions resulting in the destruction of skin, mucous membranes, and the ocular surface. A similar, more severe form of the disorder included in this spectrum is toxic epidermal necrolysis (TEN). Approximately 35% of patients suffer chronic sequelae such as vascularization, corneal scarring, conjunctival inversion to the cornea, keratinization, symblepharon, scarring of the palpebral conjunctiva, trichiasis, and severe dry eye. We focused on 80 Indian patients with SJS/TEN with severe ocular complications (SOC) and investigated the association of alleles at *HLA -A, HLA-B* and *HLA-C* loci; the controls were 50 healthy Indian volunteers. Genotyping at *HLA-A, HLA-B*, and *HLA-C* loci showed a significant positive association with HLA-A*33:03, HLA-B*44:03, and HLA-C*07:01 alleles, and a significant negative association with HLA-B*57:01 and HLA-C*06:02. This indicates that HLA-A*33:03, HLA-B*44:03 and HLA-C*07:01 are risk alleles, and HLA-B*57:01 and HLA-C*06:02 are protective alleles in this population. We also found that the haplotypes consisting of HLA-B*44:03 and HLA-C*07:01 were strongly associated with SJS/TEN with SOC in our Indian population (p = 1.1 × 10^−7^, odds ratio = 11.0). Describing the association of the haplotype could facilitate the understanding of increased risk factors for developing SJS/TEN with SOC.

## Introduction

Stevens Johnson syndrome (SJS) is part of a spectrum of adverse drug reactions resulting in the destruction of skin, mucous membranes, and the ocular surface via immune-mediated mechanisms leading to cell death by apoptosis/necrosis. A similar, more severe form of the disorder included in this spectrum is toxic epidermal necrolysis (TEN). Although the incidence of both SJS and TEN is low (approximately 2–6 cases/10^6^/year), the morbidity and mortality rate is high^1^. Epidermal detachment ranges from mild [1–10% of the total body surface area (TBSA)] in SJS to severe (>30% of TBSA) in TEN. The intermediate range of 10–30% of TBSA involvement is considered to reflect moderate severity and represents the SJS-TEN overlap. A variety of drugs such as antibiotics and non-steroidal anti-inflammatory drugs (NSAID), including cold medications and anticonvulsants, are implicated in the elicitation of SJS/TEN^2^. A fraction of SJS/TEN patients suffered microbial infections such as mycoplasma or virus infection^3^. The initial manifestations of SJS/TEN include fever, stinging in the eyes, and pain in swallowing; they are followed by the development of cutaneous lesions that can involve the entire body. The lesions appear initially as erythematous purple-red patches. With the onset of epidermal necrosis, the epidermis detaches from the underlying dermis and blisters arise due to fluid accumulation in the space between the dermis and epidermis. In most SJS/TEN patients, besides the conjunctiva, eye lids, and cornea, oral and genital mucosal tissues are involved.

Common acute ocular manifestations are bilateral severe conjunctivitis, pseudomembrane, and corneal and conjunctival epithelial defects^4^. During the acute phase of SJS/TEN, approximately 50% of patients present with severe ocular involvement^5^.

Common chronic ocular manifestations include vascularization, corneal scarring, conjunctival inversion to the cornea, keratinization, symblepharon, scarring of the palpebral conjunctiva, trichiasis, and severe dry eye^6^. Chronic eye symptoms are red eye, dry eye, pain, itching, grittiness, heavy eyelid, foreign body sensation, decreased vision, a burning sensation, photophobia, and diplopia; they are seen in approximately 35% of patients^3^.

Significant risk factors for ocular involvement in SJS include exposure to drugs such as NSAIDs and cold medications^5,7^. About 80% of Japanese- and 53% of Brazilian SJS/TEN with SOC patients had taken cold medicines^7–9^.

Genetic studies involved case-control studies aimed at identifying associations between SJS/TEN with SOC and genetic loci involved in immune response pathways and the detoxication of drugs and xenobiotics. Alleles of various human leukocyte antigen (HLA) loci were the focus of genetic association studies of SJS/TEN in different populations and ethnic differences in genetic associations have been proposed. HLA antigens associated with SJS with ocular complications include *HLAB12* or its sub-group *HLABw44* in Caucasians^10,11^; an association has also been reported in a French population with TEN^12^. Elsewhere we reported that in Japanese patients with SJS/TEN with SOC, the disease was associated with specific alleles at various HLA loci including *HLA-* A* *02:06* ^13^ and that HLA-B*44:03 was an independent risk allele for cold-medicine-induced SJS^14^. Our study across different ethnic groups with SJS/TEN with SOC suggested a significant association between HLAB*44:03 and HLA-A*02:06 alleles and SJS/TEN with SOC; in fact, we detected specificity in the associated alleles in different ethnic groups^15^.

The association of HLA-B*5801 with allopurinol-induced SJS/TEN was demonstrated in Han Chinese-^16^, European-^17^, and Japanese patients^18^. A striking observation in relation to HLA markers in Han-Chinese SJS patients is the association of carbamazepine (CBZ) and *HLA-* B*15:*02* ^19^; the predicted specificity and sensitivity for this allele as a marker for CBZ-SJS in this ethnic group was 97% and 100%, respectively. On the other hand, allopurinol-induced SJS/TEN was rarely associated with SOC^20^ and only 5% of Japanese SJS/TEN with SOC patients had taken anti-epilepsy drugs such as CBZ^8^.

In the current investigation we focused on Indian patients with SJS/TEN with SOC. We examined genetic factors by investigating the association of alleles at *HLA-A, HLA-B*, and *HLA-C* loci.

## Methods

Our study was approved by the institutional review board of the L.V. Prasad Eye Institute and Kyoto Prefectural University of Medicine. All experimental procedures were conducted in accordance with the principles set forth in the Helsinki Declaration. The purpose of the experimental protocols was explained to all participants and their prior written informed consent was obtained.

### Patients

Between July 2012 and June 2014, 80 patients with SJS/TEN with SOC (43 males, 37 females, age range 5–63 years, mean age 28.1 ± 14.2 years) were recruited at the L.V. Prasad Eye Institute. At the time of this study, their age ranged from 10–50 years. All were in the chronic stage of the disease; the diagnosis of SJS/TEN with SOC was based on a confirmed history of acute-onset high fever, serious mucocutaneous manifestations with skin eruptions, and the involvement of at least 2 mucosal sites, including the oral cavity and ocular surface in the acute stage. In the chronic stage there were the ocular previously reported manifestations such as vascularization, corneal scarring, conjunctival inversion to the cornea, keratinization, symblepharon, scarring of the palpebral conjunctiva, trichiasis, and severe dry eye^14,21–24^. The 50 normal Indian controls (27 males, 23 females, median age 36.0 ± 11.4 years) were also recruited at the L.V. Prasad Eye Institute; none had a history of SJS/TEN or related conditions or a history of cutaneous drug reactions.

### Samples

Blood samples were collected by venipuncture from all study participants. DNA was extracted from blood leukocytes by the phenol chloroform method and assessed spectrophotometrically.

### HLA typing

Genotyping for *HLA* alleles was performed by subjecting DNA samples to polymerase chain reaction (PCR) assay. This was followed by hybridization to simple-sequence oligonucleotides using a bead-based genotyping kit (Wakunaga, Hiroshima, Japan)^9,13–15,25,26^. Briefly, target DNA was PCR-amplified with biotinylated primers specifically designed for amplified exons 2 and 3 of HLA-A, HLA-B, and HLA-C genes and the PCR amplicon was denatured and hybridized to complementary oligonucleotide probes immobilized on fluorescent-coded microsphere beads. At the same time, the biotinylated PCR product was labeled with phycoerytrin-conjugated streptavidin and immediately examined with Luminex 100 (Luminex, Austin, TX, USA). Genotype determination and data analysis were performed automatically using WAKFLOW typing software (Wakunaga) according to the manufacturer’s instructions.

### Data analysis

We compared the carrier frequency and the gene frequency of individual *HLA* alleles in the patients and controls based on the dominant model using the Fisher exact test (JMP version.11 software; SAS Institute Japan Ltd., Tokyo, Japan). The odds ratio (OR) with a 95% confidence interval (CI) was calculated with Fisher’s exact test (JMP version 11 software). Significance levels were corrected with Bonferroni correction for multiple comparisons.

## Results and Discussion

As shown in Supplementary Table 1, genotyping of the 80 Indian patients and 50 controls at *HLA-A, HLA-B*, and *HLA-C* loci showed significant positive associations with HLA-A*33:03, HLA-B*44:03 and HLA-C*07:01 alleles, and significant negative associations with HLA-B*57:01 and HLA-C*06:02 (Table 1), suggesting that the association-positive alleles (HLA-A*33:03, HLA-B*44:03 and HLA-C*07:01 alleles) are risk alleles, while HLA-B*57:01 and HLA-C*06:02 are protective alleles in this population. Alleles of other HLA loci we investigated revealed no detectable association with SJS (Supplementary Table 1). Our study thus confirms an association of HLA-*B44:03 alleles in Indian patients with SJS/TEN with SOC, and first documents an association of HLA alleles HLA-A*33:03, HLA-B*57:01, HLA-C*06:02, and HLA-C*07:01 in an Indian population.

**Table 1:** HLA alleles associated with indian SJS/TEN with SOC.

HLA	Carrier frequency					Gene frequency				
	Case	Control	p-value (Fisher)	corrected p-value	odd’s ratio (95%Cl)	Case	Control	p-value (Fisher)	corrected p-value	odd’s ratio (95%Cl)
A*33:03	46.3% (37/80)	20.0% (10/50)	**2.7.E-03**	**3.2.E-02**	3.4 (1.5–7.8)	29.4% (47/160)	13% (13/100)	**2.4.E-03**	**2.9.E-02**	2.8 (1.4–5.5)
B*44:03	62.5% (50/80)	12.0% (6/50)	**7.3E-09**	**1.1.E-07**	12.2 (4.7–32.1)	43.1% (69/160)	7% (7/100)	**7.9.E-11**	**1.3E-09**	10.1 (4.4–23.1)
B*57:01	1.3% (1/80)	20.0% (10/50)	**3.0.E-04**	**4.4.E-03**	0.05 (0.006–0.4)	0.6% (1/160)	10% (10/100)	**3.9.E-04**	**6.3.E-03**	0.06 (0.007–0.5)
C*06:02	5.0% (4/80)	28.0% (14/50)	**4.0.E-04**	**5.6.E-03**	0.1 (0.04–0.4)	2.5% (4/160)	15% (15/100)	**3.0.E-04**	**4.2.E-03**	0.1 (0.05–0.5)
C*07:01	58.8% (47/80)	18.0% (9/50)	**4.4.E-06**	**6.1.E-05**	6.5 (2.8–15.1)	40.6% (65/160)	10% (10/100)	**4.9.E-08**	**6.9E-07**	6.2 (3.0–12.7)

The corrected p value indicates the p value after correction for multiple comparisons (12 for HLA-A, 15 for HLA-B, 14 for HLA-C).

Although detailed information on drugs used and infections was not available for all patients, we were able to confirm that 23 patients presented with the onset of SJS/TEN after taking cold medicines. HLA analysis of these 23 samples and of 50 control samples showed a significant positive association with HLA-B*44:03 and HLA-C*07:01 alleles and a significant negative association with HLA-C*06:02 (Supplementary Table 2).

In addition to HLA-B*44:03, we identified HLA-C*07:01 as a risk factor for cold medicine-related SJS/TEN with SOC in the current study population and HLA-C*06:02 as a strongly protective HLA allele.

HLA-A*33:03, HLA-B*44:03, and HLA-C*07:01 constitute a haplotype; 30 of our 80 SJS/TEN with SOC patients (37.5%) and only 4 of the 50 controls (8%) manifested this haplotype (p = 0.00017, OR = 6.9), as did 10 of the 23 patients (43.5%) whose CM-SJS/TEN with SOC we thought to be cold-medicine-related (p = 0.00079, OR = 8.8). Moreover, 44 of the 80 patients (55%) harbored the HLA-B*44:03 and HLA-C*07:01 haplotype (which include HLA-A*33:03, HLA-B*44:03, and HLA-C*07:01 haplotype) (p = 1.1 × 10^−7^, OR = 11.0), as did 12 of the 23 (52.2%) patients whose CM-SJS/TEN with SOC we attributed to cold medicines (p = 0.00018, OR = 9.8), and 5 of the 50 controls. This suggests strongly that the HLA-B*44:03, HLA-C*07:01 haplotype is a strong risk factor for SJS/TEN with SOC. Among the 50 controls, 10 harbored the protective HLA-B*57:01 allele and 14 (28%) were positive for HLA-C*06:02. HLA-B*57:01 was detected in only one of the 80 patients with SJS/TEN with SOC (1.3%) and HLA-C*06:02 in 4 (5%). As none of the 23 patients with cold-medicine-related CM-SJS/TEN with SOC manifested HLA-B*57:01 or HLA-C*06:02, we posit that these alleles are strongly protective factors for SJS/TEN with SOC.

In summary, we first report that in our Indian population, the haplotype comprised of HLA-B*44:03 and HLA-C*07:01 is strongly associated with SJS/TEN with SOC. Our findings also suggest that HLA-B*57:01 and HLA-C*06:02 are protective against this disease.

## Electronic supplementary material

Supplementary Tables
