# Supplementary material for: Association of Human Leukocyte Antigen Class 1 genes with Stevens Johnson Syndrome with severe ocular complications in an Indian population
Source: Sci Rep. 2017 Nov 21;7:15960. doi: 10.1038/s41598-017-15965-7 (PMC5698496; doi:10.1038/s41598-017-15965-7)
Supplement: Supplementary file 1 — Supplementary Tables [file 41598_2017_15965_MOESM1_ESM.doc]

**Association of Human Leukocyte Antigen Class 1 genes with Stevens Johnson Syndrome with severe ocular complications**

**in an Indian population**

Chitra Kannabiran^1^*, Mayumi Ueta^2#^*, Virender Sangwan^3^, Varsha Rathi^3^, Sayan Basu^3^, Katsushi Tokunaga^4^, Shigeru Kinoshita^2^

^1^Kallam Anji Reddy Molecular Genetics Laboratory, Prof Brien Holden Eye Research Centre; Tej Kohli Cornea Institute

^2^Department of Frontier Medical Science and Technology for Ophthalmology, Kyoto Prefectural University of Medicine, Kyoto, Japan

^3^L.V. Prasad Eye Institute, Kallam Anii Reddy Campus, Banjara Hills, Hyderabad, 500034, India

^4^Department of Human Genetics, Graduate School of Medicine, University of Tokyo, Tokyo, Japan

*First authors, Chitra Kannabiran and Mayumi Ueta contributed equally to the work

^#^**Corresponding author:** Mayumi Ueta

Department of Frontier Medical Science and Technology for Ophthalmology, Kyoto Prefectural University of Medicine

465 Kajiicho, Hirokoji, Kawaramachi, Kamigyoku, Kyoto 602-0841, Japan

Phone: 81-75-251-5578; Fax: 81-75-251-5663, E-mail: mueta@koto.kpu-m.ac.jp

| Supplementary Table 1 | |  |  |  |  |  |  |  |  |  |
| --- | --- | --- | --- | --- | --- | --- | --- | --- | --- | --- |
| a. HLA-A |  |  |  |  |  |  |  |  |  |  |
| HLA | Carrier frequency | | | | | Gene frequency | | | | |
|  | Case | Control | p-value (Fisher) | corrected  p-value | odd's ratio (95%Cl) | Case | Control | p-value (Fisher) | corrected  p-value | odd's ratio (95%Cl) |
|  |  |  |  |  |  |  |  |  |  |  |
| A*01:01 | 22.5%(18/80) | 28%(14/50) | 0.53 |  |  | 12.5%(20/160) | 15%(15/100) | 0.58 |  |  |
| A*02:01 | 7.5%(6/80) | 4%(2/50) | 0.71 |  |  | 3.8%(6/160) | 2%(2/100) | 0.71 |  |  |
| A*02:06 | 3.8%(3/80) | 6%(3/50) | 0.68 |  |  | 1.9%(3/160) | 3%(3/100) | 0.68 |  |  |
| A*02:11 | 6.3%(5/80) | 14%(7/50) | 0.21 |  |  | 3.1%(5/160) | 7%(7/100) | 0.22 |  |  |
| A*03:01 | 7.5%(6/80) | 8%(4/50) | 1.00 |  |  | 3.8%(6/160) | 4%(4/100) | 1.00 |  |  |
| A*11:01 | 25%(20/80) | 30%(15/50) | 0.55 |  |  | 15%(24/160) | 17%(17/100) | 0.73 |  |  |
| A*24:02 | 12.5%(10/80) | 22%(11/50) | 0.22 |  |  | 6.3%(10/160) | 11%(11/100) | 0.24 |  |  |
| A*26:01 | 10%(8/80) | 16%(8/50) | 0.41 |  |  | 5%(8/160) | 8%(8/100) | 0.43 |  |  |
| A*32:01 | 3.8%(3/80) | 12%(6/50) | 0.09 |  |  | 1.9%(3/160) | 6%(6/100) | 0.091 |  |  |
| A*33:03 | 46.3%(37/80) | 20%(10/50) | **2.7.E-03** | **3.2.E-02** | **3.4(1.5-7.8)** | 29.4%(47/160) | 13%(13/100) | **2.4.E-03** | **2.9.E-02** | **2.8 (1.4-5.5)** |
| A*68:01 | 18.8%(15/80) | 10%(5/50) | 0.22 |  |  | 9.4%(15/160) | 6%(6/100) | 0.36 |  |  |
| others | 15.0%(12/80) | 16%(8/50) | 1 |  |  | 8.1%(13/160) | 8%(8/100) | 1 |  |  |
| Corrected P is P after correction for multiple (12) comparisons. | | | | |  |  |  |  |  |  |
|  |  |  |  |  |  |  |  |  |  |  |
| a. HLA-B |  |  |  |  |  |  |  |  |  |  |
| HLA | Carrier frequency | | | | | Gene frequency | | | | |
|  | Case | Control | p-value (Fisher) | corrected  p-value | odd's ratio (95%Cl) | Case | Control | p-value (Fisher) | corrected  p-value | odd's ratio (95%Cl) |
|  |  |  |  |  |  |  |  |  |  |  |
| B*07:02 | 3.8%(3/80) | 6%(3/50) | 0.68 |  |  | 1.9%(3/160) | 3%(3/100) | 0.68 |  |  |
| B*07:05 | 3.8%(3/80) | 18%(9/50) | **1.0.E-02** |  |  | 1.9%(3/160) | 9%(9/100) | **1.2.E-02** |  |  |
| B*08:01 | 8.8%(7/80) | 2%(1/50) | 0.152 |  |  | 4.4%(7/160) | 1%(1/100) | 0.16 |  |  |
| B*13:01 | 7.5%(6/80) | 2%(1/50) | 0.25 |  |  | 3.8%(6/160) | 1%(1/100) | 0.26 |  |  |
| B*15:02 | 5%(4/80) | 2%(1/50) | 0.65 |  |  | 2.5%(4/160) | 1%(1/100) | 0.65 |  |  |
| B*18:01 | 1.3%(1/80) | 8%(4/50) | 0.072 |  |  | 0.6%(1/160) | 4%(4/100) | 0.074 |  |  |
| B*35:01 | 8.8%(7/80) | 8%(4/50) | 1.0 |  |  | 4.4%(7/160) | 4%(4/100) | 1.0 |  |  |
| B*35:03 | 12.5%(10/80) | 12%(6/50) | 1.0 |  |  | 6.3%(10/160) | 6%(6/100) | 1.0 |  |  |
| B*40:06 | 12.5%(10/80) | 28%(14/50) | **3.6.E-02** |  |  | 6.9%(11/160) | 14%(14/100) | 0.082 |  |  |
| B*44:03 | 62.5%(50/80) | 12%(6/50) | **7.3E-09** | **1.1.E-07** | **12.2 (4.7-32.1)** | 43.1%(69/160) | 7%(7/100) | **7.9.E-11** | **1.3E-09** | **10.1 (4.4-23.1)** |
| B*51:01 | 12.5%(10/80) | 10%(5/50) | 0.78 |  |  | 6.3%(10/160) | 5%(5/100) | 0.79 |  |  |
| B*52:01 | 7.5%(6/80) | 12%(6/50) | 0.53 |  |  | 3.8%(6/160) | 7%(7/100) | 0.26 |  |  |
| B*57:01 | 1.3%(1/80) | 20%(10/50) | **3.0.E-04** | **4.4.E-03** | **0.05 (0.006-0.4)** | 0.6%(1/160) | 10%(10/100) | **3.9.E-04** | **6.3.E-03** | **0.06 (0.007-0.5)** |
| B*58:01 | 6.3%(5/80) | 8%(4/50) | 0.73 |  |  | 3.1%(5/160) | 4%(4/100) | 0.74 |  |  |
| otheres | 21.3%(17/80) | 46%(23/50) | **3.6.E-03** |  |  | 10.6%(17/160) | 24%(24/100) | **5.1.E-03** |  |  |
| Corrected P is P after correction for multiple (15 ) comparisons. | | | | |  |  |  |  |  |  |
|  |  |  |  |  |  |  |  |  |  |  |
| c. HLA-C |  |  |  |  |  |  |  |  |  |  |
| HLA | Carrier frequency | | | | | Gene frequency | | | | |
|  | Case | Control | p-value (Fisher) | corrected  p-value | odd's ratio (95%Cl) | Case | Control | p-value (Fisher) | corrected  p-value | odd's ratio (95%Cl) |
|  |  |  |  |  |  |  |  |  |  |  |
| C*01:02 | 2.5%(2/80) | 10%(5/50) | 0.11 |  |  | 1.3%(2/160) | 5%(5/100) | 0.11 |  |  |
| C*03:02 | 6.3%(5/80) | 8%(4/50) | 0.73 |  |  | 3.1%(5/160) | 4%(4/100) | 0.74 |  |  |
| C*04:01 | 21.3%(17/80) | 32%(16/50) | 0.21 |  |  | 11.9%(19/160) | 16%(16/100) | 0.36 |  |  |
| C*04:03 | 6.3%(5/80) | 2%(1/50) | 0.41 |  |  | 3.1%(5/160) | 1%(1/100) | 0.41 |  |  |
| C*06:02 | 5%(4/80) | 28%(14/50) | **4.0.E-04** | **5.6.E-03** | **0.1 (0.04-0.4)** | 2.5%(4/160) | 15%(15/100) | **3.0.E-04** | **4.23.E-03** | **0.1 (0.05-0.5)** |
| C*07:01 | 58.8%(47/80) | 18%(9/50) | **4.4.E-06** | **6.1.E-05** | **6.5 (2.8-15.1)** | 40.6%(65/160) | 10%(10/100) | **4.9.E-08** | **6.92E-07** | **6.2 (3.0-12.7)** |
| C*07:02 | 21.3%(17/80) | 28%(14/50) | 0.40 |  |  | 10.6%(17/160) | 14%(14/100) | 0.44 |  |  |
| C*08:01 | 5%(4/80) | 2%(1/50) | 0.65 |  |  | 2.5%(4/160) | 1%(1/100) | 0.65 |  |  |
| C*12:02 | 10%(8/80) | 16%(8/50) | 0.41 |  |  | 5%(8/160) | 8%(8/100) | 0.43 |  |  |
| C*12:03 | 2.5%(2/80) | 6%(3/50) | 0.37 |  |  | 1.3%(2/160) | 3%(3/100) | 0.38 |  |  |
| C*14:02 | 3.8%(3/80) | 8%(4/50) | 0.43 |  |  | 1.9%(3/160) | 4%(4/100) | 0.43 |  |  |
| C*15:02 | 16.3%(13/80) | 22%(11/50) | 0.49 |  |  | 8.1%(13/160) | 11%(11/100) | 0.51 |  |  |
| C*16:02 | 3.8%(3/80) | 4%(2/50) | 1.00 |  |  | 1.9%(3/160) | 2%(2/100) | 1.00 |  |  |
| others | 12.5%(10/80) | 10%(5/50) | 0.78 |  |  | 6.3%(10/160) | 6%(6/100) | 1.00 |  |  |
| Corrected P is P after correction for multiple (14) comparisons. | | | | |  |  |  |  |  |  |

| Supplementary Table2 | |  |  |  |  |  |  |  |  |  |
| --- | --- | --- | --- | --- | --- | --- | --- | --- | --- | --- |
| a. HLA-A | |  |  |  |  |  |  |  |  |  |
| HLA | Carrier frequency | | | | | Gene frequency | | | | |
|  | Case | Control | p-value (Fisher) | corrected  p-value | odd's ratio (95%Cl) | Case | Control | p-value (Fisher) | corrected  p-value | odd's ratio (95%Cl) |
|  |  |  |  |  |  |  |  |  |  |  |
| A*01:01 | 17.4%(4/23) | 28%(14/50) | 0.39 |  |  | 8.7%(4/46) | 15%(15/100) | 0.43 |  |  |
| A*02:11 | 8.7%(2/23) | 14%(7/50) | 0.71 |  |  | 4.3%(2/46) | 7%(7/100) | 0.72 |  |  |
| A*03:01 | 4.3%(1/23) | 8%(4/50) | 1.00 |  |  | 2.2%(1/46) | 4%(4/100) | 1.00 |  |  |
| A*11:01 | 21.7%(5/23) | 30%(15/50) | 0.58 |  |  | 13%(6/46) | 17%(17/100) | 0.63 |  |  |
| A*24:02 | 17.4%(4/23) | 22%(11/50) | 0.76 |  |  | 8.7%(4/46) | 11%(11/100) | 0.78 |  |  |
| A*26:01 | 4.3%(1/23) | 16%(8/50) | 0.26 |  |  | 2.2%(1/46) | 8%(8/100) | 0.27 |  |  |
| A*32:01 | 0%(0/23) | 12%(6/50) | 0.17 |  |  | 0%(0/46) | 6%(6/100) | 0.18 |  |  |
| A*33:03 | 43.5%(10/23) | 20%(10/50) | 0.05 |  |  | 32.6%(15/46) | 13%(13/100) | **7.1.E-03** |  |  |
| A*68:01 | 21.7%(5/23) | 10%(5/50) | 0.27 |  |  | 10.9%(5/46) | 6%(6/100) | 0.32 |  |  |
| others | 34.8%(8/23) | 26%(13/50) | 0.58 |  |  | 17.4%(8/46) | 13%(13/100) | 0.61 |  |  |
| Corrected P is P after correction for multiple (10) comparisons. | | | | | |  |  |  |  |  |
|  |  |  |  |  |  |  |  |  |  |  |
| b. HLA-B | |  |  |  |  |  |  |  |  |  |
| HLA | Carrier frequency | | | | | Gene frequency | | | | |
|  | Case | Control | p-value (Fisher) | corrected  p-value | odd's ratio (95%Cl) | Case | Control | p-value (Fisher) | corrected  p-value | odd's ratio (95%Cl) |
|  |  |  |  |  |  |  |  |  |  |  |
| B*07:05 | 4.3%(1/23) | 18%(9/50) | 0.16 |  |  | 2.2%(1/46) | 9%(9/100) | 0.17 |  |  |
| B*15:02 | 17.4%(4/23) | 2%(1/50) | **3.2.E-02** |  |  | 8.7%(4/46) | 1%(1/100) | **3.4.E-02** |  |  |
| B*35:01 | 8.7%(2/23) | 8%(4/50) | 1.00 |  |  | 4.3%(2/46) | 4%(4/100) | 1.00 |  |  |
| B*35:03 | 17.4%(4/23) | 12%(6/50) | 0.72 |  |  | 8.7%(4/46) | 6%(6/100) | 0.73 |  |  |
| B*40:06 | 4.3%(1/23) | 28%(14/50) | 0.03 |  |  | 2.2%(1/46) | 14%(14/100) | **3.7.E-02** |  |  |
| B*44:03 | 60.9%(14/23) | 12%(6/50) | **3.3.E-05** | **3.3.E-04** | **11.4 (3.5-37.7)** | 47.8%(22/46) | 7%(7/100) | **4.1.E-08** | **4.1.E-07** | **12.2 (4.7-31.9)** |
| B*51:01 | 8.7%(2/23) | 10%(5/50) | 1.00 |  |  | 4.3%(2/46) | 5%(5/100) | 1.00 |  |  |
| B*52:01 | 4.3%(1/23) | 12%(6/50) | 0.42 |  |  | 2.2%(1/46) | 7%(7/100) | 0.44 |  |  |
| B*57:01 | 0%(0/23) | 20%(10/50) | **2.5.E-02** |  |  | 0%(0/46) | 10%(10/100) | **3.1.E-02** |  |  |
| others | 39.1%(9/23) | 72%(36/50) | **1.0.E-02** |  |  | 19.6%(9/46) | 37%(37/100) | **3.7.E-02** |  |  |
| Corrected P is P after correction for multiple (10) comparisons. | | | | | |  |  |  |  |  |
|  |  |  |  |  |  |  |  |  |  |  |
| c. HLA-C | |  |  |  |  |  |  |  |  |  |
| HLA | Carrier frequency | | | | | Gene frequency | | | | |
|  | Case | Control | p-value (Fisher) | corrected  p-value | odd's ratio (95%Cl) | Case | Control | p-value (Fisher) | corrected  p-value | odd's ratio (95%Cl) |
|  |  |  |  |  |  |  |  |  |  |  |
| C*01:02 | 0%(0/23) | 10%(5/50) | 0.17 |  |  | 0%(0/46) | 5%(5/100) | 0.33 |  |  |
| C*04:01 | 21.7%(5/23) | 32%(16/50) | 0.42 |  |  | 13%(6/46) | 16%(16/100) | 0.80 |  |  |
| C*06:02 | 0%(0/23) | 28%(14/50) | **3.3.E-03** | **3.7.E-02** | **0.05 (0.03-0.9)** | 0%(0/46) | 15%(15/100) | **2.9.E-03** | **2.7.E-02** | **0.06 (0.03-1.0)** |
| C*07:01 | 60.9%(14/23) | 18%(9/50) | **7.9.E-04** | **8.7.E-03** | **7.1 (2.3-21.4)** | 47.8%(22/46) | 10%(10/100) | **8.8.E-07** | **7.9.E-06** | **8.3 (3.4-19.7)** |
| C*07:02 | 13%(3/23) | 28%(14/50) | 0.24 |  |  | 6.5%(3/46) | 14%(14/100) | 0.27 |  |  |
| C*08:01 | 17.4%(4/23) | 2%(1/50) | **3.2.E-02** |  |  | 8.7%(4/46) | 1%(1/100) | **3.4.E-02** |  |  |
| C*12:02 | 8.7%(2/23) | 16%(8/50) | 0.49 |  |  | 4.3%(2/46) | 8%(8/100) | 0.51 |  |  |
| C*15:02 | 0%(0/23) | 22%(11/50) | **1.3.E-02** |  |  | 0%(0/46) | 11%(11/100) | **1.7.E-02** |  |  |
| others | 34.8%(9/23) | 30%(19/50) | 1.00 |  |  | 19.6%(9/46) | 20%(20/100) | 1.00 |  |  |
| Corrected P is P after correction for multiple (9) comparisons. | | | | | |  |  |  |  |  |
